# Effects on preventing mother-to-child transmission of syphilis and associated adverse pregnant outcomes: a longitudinal study from 2001 to 2015 in Shanghai, China

**DOI:** 10.1186/s12879-017-2721-1

**Published:** 2017-09-18

**Authors:** Yang Li, Liping Zhu, Li Du, Lingxiao Qu, Weili Jiang, Biao Xu

**Affiliations:** 1 Department of Epidemiology, School of Public Health Fudan University, Shanghai, China; 2 Key Laboratory of Public Health Safety (Ministry of Education), Shanghai, China; 3 Shanghai Center for Women and Children’s Health, Shanghai, China; 4 Department of Public Health Sciences (Global Health/IHCAR), Karolinska Institutet, Stockholm, Sweden

**Keywords:** Syphilis, Mother-to-child transmission, Adverse pregnant outcomes, Epidemiology

## Abstract

**Background:**

Maternal syphilis is a health threat to both the pregnant women and the children. This study aimed to delineate the longitudinal trend of maternal syphilis and burden of associated adverse pregnant outcomes (APOs) in Shanghai from 2001 to 2015; and to evaluate the effects of preventing mother-to-child transmission (PMTCT) of syphilis in Shanghai with regard to service coverage and APOs averted.

**Methods:**

PMTCT program of syphilis has been implemented since 2001. Municipal and national PMTCT surveillance data were used in analysis. By using WHO estimation model, the burden of associated APOs and APOs averted were estimated. The differences in access to antenatal care and PMTCT services between resident and non-resident pregnant women were analyzed.

**Results:**

The prevalence of seropositivity for maternal syphilis in Shanghai ranged from 0.20% to 0.38% during 2001–2015. The treatment rate varied from 69.8% to 96.8% and remained 83.6% in 2015. Under the PMTCT program, 2163 APOs had been averted during the 15-year period, including 852(39.4%) early fetal loss/stillbirth, 356(16.4%) neonatal death, 190(8.8%) prematurity or low birth weight, and 765(35.4%) clinical evidence of congenital syphilis. Compared with the residents, the non-resident pregnant women had a higher prevalence of syphilis (1.2‰ vs. 2.5‰) and contributed to 81.7% of the syphilis associated APOs in 2015.

**Conclusion:**

Screening of maternal syphilis has reached a full coverage both in residents and non-residents. Large numbers of APOs has been averted attributing to the PMTCT program. More attentions should be paid to those vulnerable non-resident pregnant women and tailored interventions including health education, PMTCT promotion and point of care should be given to maximize the effects of PMTCT in Shanghai.

## Background

Syphilis, a severe sexually transmitted disease, remains a global public health problem with an estimated 17.7 million prevalent cases at the age of 15–49 years in 2012 [1–3]. Pregnant women with syphilis can transmit the infection to their fetus, causing congenital syphilis and other adverse pregnant outcomes (APOs), especially when there is no treatment or no adequate treatment [4]. In early maternal syphilis, the mother-to-child transmission can be up to 80% [5]. According to the World Health Organization (WHO), every year, there are 2 million pregnant women infected with syphilis, and about 1.2 million mother-to-child transitions happened [6]. Globally, it was estimated that nearly 0.5 million APOs were caused by maternal syphilis, including stillbirths, neonatal deaths, preterm or low birth weight (LBW), and congenital syphilis. About 66% of these APOs occurred in women without receiving syphilis screening and treatment during pregnancy [7]. In China, according to national surveillance, the prevalence of maternal syphilis among pregnant women increased from 0.20% in 2011 to 0.24% in 2013 [8].

Congenital syphilis could be prevented through antenatal screening and adequate treatment [5]. Many studies have proved that the intervention on preventing mother to child transmission (PMTCT) of syphilis are feasible and highly cost-effective [9–11]. In 2007, WHO launched the initiative for the global elimination of congenital syphilis (ECS), with the goal that by 2015 at least 90% of pregnant women are tested for syphilis in antenatal care (ANC) and at least 90% of seropositive pregnant women receive adequate treatment [5]. At the early days of New China, the laws and regulations against commercialized sex were enacted and sexually transmitted diseases including syphilis were almost eliminated in 1960s [12]. However, syphilis has staged a comeback and become epidemic again in China since 1980s with the rapid urbanization, sexual revolution and reemerging of commercial sex services [13, 14]. At the beginning of 21 century, PMTCT program of human immunodeficiency virus (HIV) had been initiated in China, together with a pilot PMTCT of syphilis in some cities like Shanghai and Shenzhen [15–17]. After a decade practice, in 2011, the integrated program for PMTCT of HIV, syphilis and hepatitis B virus (HBV) were formally launched by the ministry of health (MOH), China [8].

Shanghai, as one of the most developed cities in China, has attracted millions of domestic rural migrants dwelling. The population in Shanghai has been expanded from 11.0 million in 1978 to 24.3 million in 2015 consisting of 58.9% resident people and 41.1% being non-residents [18]. The reported syphilis incidence in Shanghai has also been rising from 0.48 per 100,000 in 1990 [19] to 56.32 per 100,000 in 2013 [20]. In 2015, there were 13,649 syphilis reported (23.3% were non-residents) [21]. For the prevention of maternal-to-child transmission of syphilis, the municipal PMTCT program of syphilis was launched in 2001 [22]. The PMTCT program was upgraded and adjusted for local situation in 2011 following the announcement of the national PMTCT guideline [23]. Nowadays, the program has been integrated comprehensively into the municipal maternal health care system and implemented at all the health facilities for ANC and delivery service.

This study aimed to delineate the longitudinal trend of maternal syphilis and burden of associated APOs in Shanghai from 2001 to 2015; and to evaluate the effects of PMTCT of syphilis in Shanghai with regard to service coverage and APOs averted.

## Methods

### PMTCT of syphilis in Shanghai

**Fig. 1:**
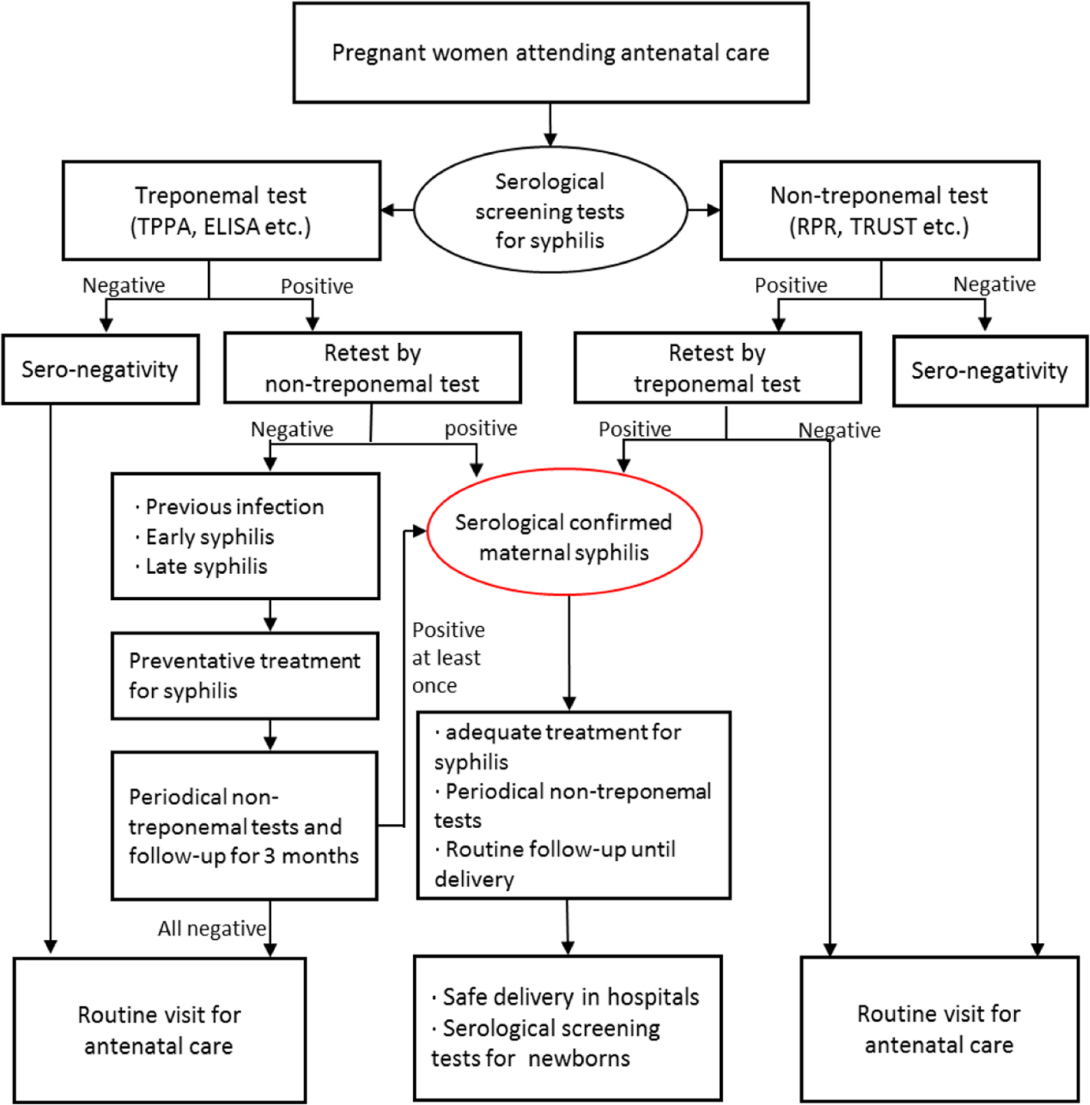
Flowchart of the PMTCT services of syphilis in Shanghai (the 2011 Version)

PMTCT program of syphilis has been set in routine antenatal care in Shanghai since 2001 covering both resident and non-resident pregnant women. At the first ANC visit, treponemal and non-treponemal serological screening should be provided to all pregnant women free of charge. Diagnosis of maternal syphilis is confirmed by both seropositive results. All diagnosed cases should be referred to “designated hospital” for sexually transmitted diseases (STDs), where the adequate treatment with penicillin and/or other antibiotics should be given immediately. The infected women should be followed up monthly with non-treponemal testing until delivery. As for pregnant women with a single-positive result, they would receive a one course preventive treatment. Serological testing should also be performed at late gestation and before delivery in order to evaluate the antibody-titer levels of syphilis and to catch those who haven’t attended a full ANC procedure (Fig. 1).

### Diagnosis of maternal syphilis

The serologic-based diagnosis of maternal syphilis uses both non-treponemal and treponemal testing. The non-treponemal test detects the non-specific antibody to reaginic antigen, including the tolulized red unheated serum test (TRUST) and rapid plasma reagin (RPR) test. The treponemal test uses the specific antigen of *T. pallidum* mainly through *T. pallidum* particle agglutination assay (TPPA) and Enzyme Linked Immunosorbent Assay (ELISA). Syphilis screening using non-treponemal test or treponemal test should be provided to pregnant women at the time of their first ANC visit. If the screening test shows a positive result, these women should be asked to have the second test for confirmatory diagnosis. Only when both serologic tests of syphilis present a positive result could the pregnant women be diagnosed as maternal syphilis.

### Data collection

Data on maternal syphilis screening and treatment in this study were distracted from the municipal and national PMTCT surveillance systems. Once diagnosed with maternal syphilis, information on demographics, history of syphilis infection and the serologic testing results of the infected women should be collected and further recorded into the PMTCT surveillance system by health providers at the ANC hospitals. Additional information about syphilis treatment, pregnant outcomes and health status of newborns should also be reported to the surveillance system. The population data used for the APOs estimation, including live-births and stillbirths etc., were collected from Shanghai antenatal care system and Shanghai health statistical yearbooks.

### Data analysis

WHO has developed an estimation model to evaluate the disease burden of syphilis in pregnancy and associated APOs, which could be applied online [24]. The methodology has been approved by the Child Health Epidemiology Research Group and used for the recent global estimates. Six parameters, i.e., number of live births, number of stillbirths, proportion of pregnant women with positive test, at least one ANC visits, syphilis screening, and adequate treatment percentage should be given to estimate the prevalence of maternal syphilis and APOs. Based on literature reviews [25], the probabilities of associated APOs occurring without effective treatment were estimated as 52% for any adverse outcome, 21% for stillbirth/early fetal death (EFD), 9% for neonatal death, 6% for prematurity and low birth weight (LBW), and 16% for congenital syphilis (CS). The effectiveness of screening and treatment with penicillin in averting above APOs were 84%, 82%, 80%, 64% and 97% respectively [26]. After setting the relative error of 5%, point estimates of burden of syphilis-related APOs can be calculated by this algorithm automatically. Meanwhile, the APOs averted by current PMTCT efforts and ECS targets can also be estimated, which could be applied to assess the reductions of syphilis associated APOs.

## Results

### Screening and treatment for maternal syphilis in Shanghai

From 2001 to 2015, about 2.8 million pregnant women received serological screening for syphilis in Shanghai. The screening rate had been rising quickly from 63.0% at onset to 95.0% in 2007, and reached a nearly full coverage of 99.6% in 2015. In total, there were 7149 maternal syphilis cases detected during the 15-year period. The highest prevalence of maternal syphilis was 0.38% in 2007, and then decreased to 0.20% in 2015. The proportion of adequate treatment among detected maternal syphilis cases was 96.8% in 2001, reduced to 69.8% in 2011 and rebounded to 83.6% in 2015.

**Fig. 2:**
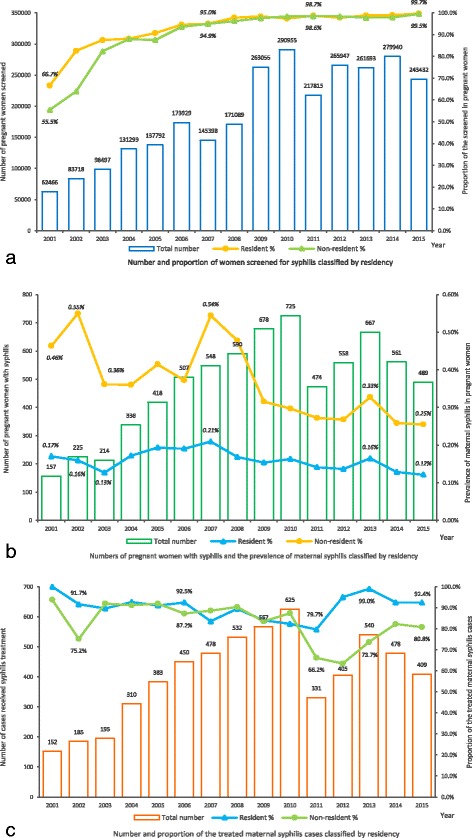
The screening and treatment for maternal syphilis in Shanghai from 2001 to 2015. **a** In this figure, the number of women screened for syphilis was presented on the bar graph in whole. The proportion was classified by residency and presented on the line graph with labels. **b** In this figure, the number of pregnant women with syphilis was presented on the bar graph in whole. The prevalence of maternal syphilis was classified by residency and presented on the line graph with labels. **c** In this figure, the number of treated maternal syphilis cases was presented on the bar graph in whole. The proportion was classified by residency and presented on the line graph with labels

Classified by resident status, the coverage of screening among non-resident pregnant women is lower than residents at the beginning, and the disparity began to shrink after 2005 and reached an almost full coverage to all pregnant women (Fig. 2a). It was found although the prevalence of maternal syphilis decreasing both in residents and non-residents, it was higher in the non-resident pregnant women (0.2%–0.6%) compared to the residents (0.1%–0.2%) (Fig. 2b). As for the treatment for maternal syphilis, no significant differences were found between residents and non-residents before 2010. However, the resident infected cases had a higher treatment percentage during 2011–2015 (from 79.7% to 92.4%) than the non-resident cases (from 66.2% to 80.8%) (Fig. 2c).

### Estimation on associated adverse outcomes for maternal syphilis

**Fig. 3:**
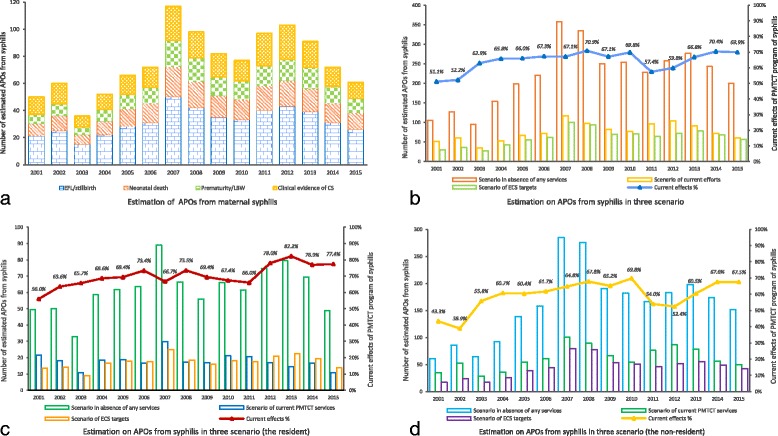
The estimation on APOs from in three scenario in Shanghai from 2001 to 2015. **a** In this figure, the estimation of APOs on each bar graph was categorized by EFL/stillbirth, neonatal death, prematurity/LBW and clinical evidence of CS. **b** In this figure, we estimated the APOs in three scenario by using the overall data including both the resident and non-resident. **c** In this figure, we estimated the APOs in three scenario happened among resident maternal syphilis cases

According to the WHO syphilis associated APOs calculation, in total, 1195 maternal syphilis associated APOs were reported during 2001 to 2015 (Fig. 3a), including 503(42.5%) early fetal loss (EFL)/stillbirth, 223(18.8%) neonatal death, 195(16.5%) prematurity or LBW, and 263(22.2%) clinical evidence of congenital syphilis. The APOs happened among non-resident maternal syphilis cases accounted for the majority of the APOs in Shanghai varied from 61.4% to 84.8% (Table 1).

**Table 1:** Estimation of Shanghai’s disease burden of syphilis associated APOs during 2001–2015

Year	EFL/Stillbirth		Neonatal death		Prematurity/LBW		Congenital Syphilis		Total APOs	
	Resident	Non-resident	Resident	Non-resident	Resident	Non-resident	Resident	Non-resident	Resident	Non-resident
2001	9	14	4	6	3	5	5	9	22(38.6%)	35(61.4%)
2002	8	21	3	9	3	7	4	15	18(25.7%)	52(74.3%)
2003	5	12	2	5	2	4	2	7	11(27.5%)	29(72.5%)
2004	8	15	4	7	3	6	4	8	18(33.3%)	36(66.7%)
2005	8	23	4	10	3	9	4	13	19(25.7%)	55(74.3%)
2006	7	25	3	11	3	10	3	14	17(21.8%)	61(78.2%)
2007	13	42	6	19	5	17	6	22	30(23.1%)	100(76.9%)
2008	8	38	3	17	3	15	3	18	18(16.8%)	89(83.2%)
2009	7	28	3	12	3	11	3	14	17(20.5%)	66(79.5%)
2010	9	23	4	11	4	10	5	11	22(28.6%)	55(71.4%)
2011	9	32	4	14	4	11	5	19	21(21.4%)	77(78.6%)
2012	7	36	3	16	3	13	2	22	16(15.5%)	87(84.5%)
2013	6	33	3	15	3	12	1	18	14(15.2%)	78(84.8%)
2014	7	24	3	11	3	10	2	12	16(22.2%)	56(77.8%)
2015	5	21	2	9	2	8	2	10	11(18.3%)	49(81.7%)

### Estimation of syphilis associated APOs averted by the PMTCT program

If there were no PMTCT services in Shanghai, it was estimated that 3301 syphilis associated APOs would have occurred from 2001 to 2015 in Shanghai (Fig. 3b), including 1333(40.4%) EFL/stillbirth, 571(17.3%) neonatal death, 381(11.5%) prematurity or LBW, and 1016(30.8%) clinical evidence of CS.

With the integrated PMTCT program, 2163 APOs has been averted during this 15-year period, including 852(39.4%) EFL/stillbirth, 356(16.4%) neonatal death, 190(8.8%) prematurity or LBW, and 765(35.4%) clinical evidence of CS. Both the resident and non-resident syphilis infected pregnant women were benefitted remarkably. It was observed that the gap between the numbers of syphilis associated APOs and ECS goals had been narrowed by years and the ECS goals was met in 2012 among resident pregnant women (Fig. 3c). Although stratified by residency, the ECS goals in non-resident syphilis infected women has yet to meet, the gap between current services and the goal had been reduced significantly in recent 5 years (Fig. 3d). The estimated effective rate of PMTCT for syphilis had been maintained at 76.9%–82.2% among resident women during recent four years.

## Discussion

The prevalence of seropositivity for maternal syphilis in Shanghai ranged from 0.20% to 0.38% during 2001–2015, which is comparable to some high-income countries (Ireland [27] 0.29% in 2005–2012, Germany [28] 0.30% in 2010). Compared with other domestic provinces in China, it was much lower (Shenzhen City [15] 0.52% in 2005, Guangzhou City [29] 0.60% in 2008, Jilin City [30] 0.58% in 2010). Based on WHO syphilis associated APOs estimation model, a large number of APOs had been prevented effectively under the current PMTCT program.

Remarkable achievements have been attained through the implementation of PMTCT. Firstly, municipal and national PMTCT programs provide guidelines for standardized clinical practice and systematic management of maternal syphilis. Secondly, “Early Warning and Management System for Pregnancy Risks” [31] in Shanghai provides routine follow-up for pregnant women who are assessed as at high risk, including maternal syphilis. Thirdly, nearly 100% coverage of antenatal care guarantees the accessibility to diagnosis and treatment for maternal syphilis. Last but not least, information system for maternal syphilis helps surveillance and follow-up.

But PMTCT program in Shanghai is still facing a persistent growth of maternal syphilis. One of the reasons for this increase was the abolition of compulsory premarital health check-up (CPH) since 2003. It was reported that the coverage of CPH dropped from 60% in 2003 to nearly 0% in 2006 [32]. A study conducted by Chen Y and his colleagues reported that the infection rate of HPV and protozoon infection increased in ANC after 2003, which should be detected earlier in CPH [33]. Moreover, although the first ANC is regulated at 1st trimester, a big part of pregnant women, especially the non-residents women, wouldn’t attend ANC until the 2nd or 3rd trimester. One study in Shanghai found that only 67.9% of pregnant women could receive early ANC before 12 GW [34]. In addition, it was observed that proportion of adequate treatment undergone a decrease during 2011–2012 when the national integrated PMTCT program was initially implemented. During the shift and integration of municipal and national PMTCT surveillance systems, some treatment and newborn follow-up information might be missing, which could lead to the underestimation of the proportion of adequate treatment. With the improvement of national PMTCT system, this indicator rebounded to 83.6% in 2015. Even so, there were still gaps between the coverage in local and the goal of ECS (90%). To improve the accessibility of syphilis treatment, Shanghai health authority appointed additional thirty-two maternity hospitals as the “designed hospitals” for maternal syphilis medical care in 2016, where infected pregnant women could receive syphilis treatment, specialized antenatal care and safe delivery at the same facility.

We found that there were disparities in maternal syphilis screening, treatment between the resident and non-resident pregnant women. Meanwhile, it was observed that the proportion of estimated APOs incurred among these non-residents increased from 61.4% in 2001 to 81.7% in 2015. Due to poor reproductive health knowledge, unfamiliar with antenatal care system and low coverage of health insurance [35–37], the non-resident cases were the vulnerable group for on time screening and adequate treatment. Furthermore, the high mobility of these women also hinders their receiving regular ANCs. A previous study of ours found that although 49.7% of the non-resident women had adequately utilized antenatal care, only 19.7% of them visited ANC during the first trimester in Shanghai [38]. Therefore, more attentions should be paid to those vulnerable non-resident pregnant women and tailored interventions including health education, PMTCT promotion and point of care should be given to maximize the effects of PMTCT effects in Shanghai, and to reduce the inequity in maternal healthcare utilization.

Based on the comprehensive surveillance system, our study is able to describe the longitudinal trend of maternal syphilis and burden of APOs in Shanghai over 15 years. Still, there are some limitations in this study. First of all, the municipal report system and national PMTCT system were experiencing an alternation in 2011–2012, thus, data from different sources had showed slight inconsistence. Secondly, the fixed parameters in WHO estimation model was based on several studied carried out in other countries, which may influence the accuracy and validity of estimation when applying to Shanghai data.

## Conclusions

This study found that screening of maternal syphilis has reached a full coverage both in residents and non-residents, and the prevalence of syphilis among pregnant women is consistently decreasing during the past 15 years. Attributing to the PMTCT program, large numbers of APOs associated with syphilis have been averted. More attentions should be paid to those vulnerable non-resident pregnant women to reduce the inequity on maternal healthcare.

## References

[1] Jenniskens F, Obwaka E, Kirisuah S, Moses S, Yusufali FM, Achola JO, Fransen L, Laga M, Temmerman M (1995). Syphilis control in pregnancy: decentralization of screening facilities to primary care level, a demonstration project in Nairobi, Kenya. Int J Gynaecol Obstet.

[2] Golden MR, Marra CM, Holmes KK (2003). Update on syphilis: resurgence of an old problem. JAMA.

[3] Newman L, Rowley J, Vander Hoorn S, Wijesooriya NS, Unemo M, Low N, Stevens G, Gottlieb S, Kiarie J, Temmerman M (2015). Global estimates of the prevalence and incidence of four curable sexually transmitted infections in 2012 based on systematic review and global reporting. PLoS One.

[4] World Health Organization. Elimination of mother-to-child transmission (EMTCT) of HIV and syphilis. http://cdrwww.who.int/hiv/pub/emtct-validation-guidance/en/. Accessed 4 June 2016. 10.2471/BLT.16.185033 PMC5096356 27821878

[5] World Health Organization. The global elimination of congenital syphilis: rationale and strategy for action. http://www.who.int/reproductivehealth/publications/rtis/9789241595858/en/. Accessed 4 June 2016.

[6] World Health Organization. Advancing MDGs 4, 5 and 6: impact of congenital syphilis elimination. http://apps.who.int/iris/bitstream/10665/70331/1/WHO_RHR_HRP_10.01_eng.pdf. Accessed 4 June 2016.

[7] Newman L, Kamb M, Hawkes S, Gomez G, Say L, Seuc A, Broutet N (2013). Global estimates of syphilis in pregnancy and associated adverse outcomes: analysis of multinational antenatal surveillance data. PLoS Med.

[8] Wang AL, Qiao YP, Wang LH, Fang LW, Wang F, Jin X, Qiu J, Wang XY, Wang Q, Wu JL (2015). Integrated prevention of mother-to-child transmission for human immunodeficiency virus, syphilis and hepatitis B virus in China. Bull World Health Organ.

[9] Hawkes S, Matin N, Broutet N, Low N (2011). Effectiveness of interventions to improve screening for syphilis in pregnancy: a systematic review and meta-analysis. Lancet Infect Dis.

[10] Kahn JG, Jiwani A, Gomez GB, Hawkes SJ, Chesson HW, Broutet N, Kamb ML, Newman LM (2014). The cost and cost-effectiveness of scaling up screening and treatment of syphilis in pregnancy: a model. PLoS One.

[11] Bristow CC, Larson E, Anderson LJ, Klausner JD (2016). Cost-effectiveness of HIV and syphilis antenatal screening: a modelling study. Sex Transm Infect.

[12] Tucker JD, Cohen MS (2011). Chinaʼs syphilis epidemic: epidemiology, proximate determinants of spread, and control responses. Curr Opin Infect Dis.

[13] Hesketh T, Zhu WX, Zhou XD (2010). Syphilis and social upheaval in China. N Engl J Med.

[14] Lin CC, Gao X, Chen X, Chen Q, Cohen MS (2006). China's Syphilis epidemic: a systematic review of seroprevalence studies. Sex Transm Dis.

[15] Hong F, Yang Y, Liu X, Feng T, Liu J, Zhang C, Lan L, Yao M, Zhou H (2014). Reduction in mother-to-child transmission of syphilis for 10 years in Shenzhen, China. Sex Transm Dis.

[16] National Center for Women and Children's Health of China CDC: China's PMTCT program of HIV (MOH-UNICEF). http://www.chinawch.org.cn/fnwsbjb. Accessed 28 Dec 2016.

[17] Zhu L, Qin M, Du L, Xie R, Wong T, Wen SW (2010). Maternal and congenital syphilis in Shanghai, China, 2002 to 2006. Int J Infect Dis.

[18] Shanghai Statistic Bureau. 2015 Shanghai Statistic Yearbook. http://www.stats-sh.gov.cn/. Accessed 28 Dec 2016.

[19] National Syphilis Epidemiology Group. Epidemiological Report on Syphilis in 1990: Surveillance data from 38 cities in China. Chin J Dermatol. 1992(4):228–31.

[20] Zhou L, Zhuang M, Ning Z, Fu J, Shen X, Gao X, Pan Q, Cheng H. Epidemiological analysis of syphilis in Shanghai, during 2005-2013. Chin J AIDS STD. 2015(04):311–3.

[21] Shanghai Municipal Commission of Health and Family Planning. Notifiable infectious disease report in Shanghai in 2015. http://www.wsjsw.gov.cn/. Accessed 28 Dec 2016.

[22] Shanghai Municipal Commission of Health and Family Planning: Shanghai municipal PMTCT program of Syphilis. http://www.wsjsw.gov.cn/. Accessed 28 Dec 2016.

[23] Shanghai Municipal Commission of Health and Family Planning: Shanghai municipal PMTCT program of HIV, Syphilis and HBV in 2011. http://www.wsjsw.gov.cn/. Accessed 28 Dec 2016.

[24] World Health Organization. Tool to estimate burden of maternal syphilis and adverse outcomes. http://www.who.int/entity/reproductivehealth/topics/rtis/syphilis/measurement_tool/en/. Accessed 27 Sept 2016.

[25] Gomez GB, Kamb ML, Newman LM, Mark J, Broutet N, Hawkes SJ (2013). Untreated maternal syphilis and adverse outcomes of pregnancy: a systematic review and meta-analysis. B World Health Organ.

[26] Pan American Health Organization. Regional iniciative for the elimination of mother-to-child transmission of HIV and congenital syphilis in Latin America and the Caribbean: regional monitoring strategy. https://www.unicef.org/lac/Regional_Monitoring_Strategy(2).pdf. Accessed 28 Sept 2016.

[27] McGettrick P, Ferguson W, Jackson V, Eogan M, Lawless M, Ciprike V, Varughese A, Coulter-Smith S, Lambert JS (2016). Syphilis serology in pregnancy: an eight-year study (2005-2012) in a large teaching maternity hospital in Dublin, Ireland. Int J STD Aids.

[28] World Health Organization. Global Health Observatory data repository. http://apps.who.int/gho/data/node.main.A1359STI?lang=en. Accessed 28 Sept 2016.

[29] Yang J, Deng L, Huang X, Yang W, Huang R, Deng X, Wang X, Ye T (2008). Epidemiological investigation of syphilitic and HIV infection in early pregnant women in Guangzhou urban center. Matern Child Health Care China.

[30] Tian X (2011). Sexually transmitted disease among pregnant women in Jilin Province. China Pract Med.

[31] Zhu L, Qing M, Shi H, Tan J, He L (2013). Initial effect of early warning and management system for pregnant risks in Shanghai. Matern Child Health Care China.

[32] Du L, Chen Y (2006). Premarital examinations in China. Matern Child Health Care China.

[33] Chen Y, Ren A, Ye R, Zheng J, Li S, Liu J, Hao L, Li Z (2008). Impacts on adverse pregnancy outcomes and relevant risk factors before and after abolish of the coercion premarital medical examination. Matern Child Health Care China.

[34] Jiao N. Study on the implementation process and effectiveness of the basic maternal and child health care services. China: Fudan University; 2014.

[35] Qi Z, Kulane A, Yi G, Xu B (2009). Knowledge and attitude on maternal health care among rural-to-urban migrant women in Shanghai, China. BMC Womens Health.

[36] Gao Y, Hong Z, Singh NS, Powelljackson T, Nash S, Min Y, Guo S, Hai F, Alvarez MM, Liu X. Progress and challenges in maternal health in western China: a countdown to 2015 national case study. Lancet Global Health. 2017; 10.1016/S2214-109X(17)30100-6 PMC5387688 28341117

[37] Long Q, Zhang TH, Xu L, Tang SL, Hemminki E (2010). Utilisation of maternal health care in western rural China under a new rural health insurance system (new co-operative medical system). Tropical Med Int Health.

[38] Zhao Q, Huang Z, Yang S, Pan J, Smith B, Xu B (2012). The utilization of antenatal care among rural-to-urban migrant women in Shanghai:a hospital-based cross-sectional study. BMC Public Health.

